# Engaging community health center advisors to identify research priorities for health equity

**DOI:** 10.1017/cts.2025.10179

**Published:** 2025-10-28

**Authors:** Nynikka R. Palmer, Michael B. Potter, Saji Mansur, Cecilia Hurtado, Maria Carbajal, Gary Bossier, Maria Echaveste, Paula Fleisher, Carlos Guerra-Sanchez, Stutee Khandelwal, Gena Lewis, Lali Moheno, Tung Nguyen, David Ofman, Kerrington Osborne, James D. Harrison

**Affiliations:** 1 Division of General Internal Medicine at Zuckerberg San Francisco General Hospital, University of California San Francisco, San Francisco, CA, USA; 2 Department of Family and Community Medicine, University of California San Francisco, San Francisco, CA, USA; 3 Clinical and Translational Science Institute, University of California San Francisco, San Francisco, CA, USA; 4 School of Medicine, University of California San Francisco, San Francisco, CA, USA; 5 HERE with Community Advisory Board, San Francisco, CA, USA; 6 Department of General Internal Medicine at Fresno, University of California San Francisco, Fresno, CA, USA; 7 Family HealthCare Network, Visalia, CA, USA; 8 Department of Pediatrics, Benioff Children’s Hospital Oakland, University of California San Francisco, Oakland, CA, USA; 9 Division of General Internal Medicine, University of California San Francisco, San Francisco, CA, USA; 10 San Francisco Community Clinic Consortium, San Francisco, CA, USA; 11 Division of Hospital Medicine, University of California San Francisco, San Francisco, CA, USA

**Keywords:** Health equity, COVID-19, community health centers, research priorities, medically underserved areas

## Abstract

**Background:**

Community health centers (CHCs) and those most burdened by disease are important partners in setting research agendas to address the needs of people who are medically underserved.

**Objectives:**

Identify and prioritize health equity-focused research priorities using a collaborative approach to community engagement of key informants.

**Methods:**

We used five stepwise phases from January 2021 to February 2023 to formulate and prioritize a set of health equity-focused research topics among CHC staff (leaders, clinicians), their key advisors (patients and community members), and researchers from academic medical centers in California. Phases included: (1) community advisory board formation, (2) key informant identification, (3) individual/small group interview guide development and administration, (4) initial health equity-focused topic categorization, and (5) in-person meeting with community advisors for final topic prioritization using nominal group technique.

**Results:**

Twenty individual or small group interviews were completed with 44 diverse participants, along with engagement from our community advisory board, which resulted in an initial list of 11 health equity-focused research topics. Ninety advisors including diverse community members, CHC staff/leaders, and researchers prioritized six overarching research topics. Final prioritized health-equity focused research topics include addressing mental health challenges, improving public’s trust in healthcare and science, healthcare delivery models to increase access and utilization, build and sustain an anti-racist healthcare system, strategies and interventions to address health misinformation, and continuing and sustaining polices based on lessons learned from COVID-19.

**Conclusions:**

Results offer future direction for community-engaged research agendas to advance health equity among medically underserved and vulnerable patient populations.

## Study Highlights

### What is the current knowledge on the topic?

Community health center (CHC) involvement in research remains challenging and uncommon. A potential way to expand research in this setting is to implement a research agenda that prioritizes the research questions that are most important to CHCs and the communities they serve. We currently do not have a community engaged perspective on what this research agenda should be.

### What question did this study address?

We aimed to identify and prioritize health equity-focused research priorities using a collaborative approach to community engagement and prioritization of research topics.

### What does this study add to our knowledge?

This study reports a prioritized set of health equity focused research topics relevant to CHCs. This includes mental health, trust, healthcare delivery, anti-racism, addressing misinformation, and sustaining lessons learned.

### How might this change clinical pharmacology or translational science?

Our findings offer important directions to advance health equity-focused research to meet the needs of medically underserved communities burdened by disease and health disparities.

## Introduction

Many research agendas emanate from academic health centers, often without any input from key advisors – including patients and community members most burdened with disease and disparities, healthcare providers, and community advocates who provide care to medically underserved communities [1]. One key lesson is how strong public collaborations among communities and health care professionals can address barriers to health care, if guided by an equity framework [2], which includes giving voice to key community advisors at research and decision-making tables. Engaging community advisors in research leads to better research processes and outcomes, builds on community interests and strengths, and combines knowledge with social change action to improve community health and eliminate health disparities [3].

Community health centers (CHCs) including federally qualified health centers and other safety-net systems deliver primary care to the most socioeconomically disadvantaged, including disproportionate numbers of people from racial and ethnic minority groups, and immigrant populations [4,5]. These healthcare environments are important partners in setting health equity research agendas to address the needs of medically underserved populations. CHCs remain an untapped wealth of knowledge, expertise, and understanding of community and patient needs [1,5,6]. Despite high levels of interest in expanding CHC involvement in research, engaging CHCs and the communities they serve to partner in research remains uncommon. More recent studies have set out to advance a collaborative research culture that promotes knowledge exchange and fosters health equity efforts [7]. However, many studies are not guided by an equity framework and persist to exclude key informants to establish or carry out research priorities that reflect voices from patients/community members [8–10] and healthcare professionals [11].

Undoubtedly, community-engaged research with CHCs has its challenges [1,7,12]. CHCs are focused on improving quality of care for the medically underserved patients they serve, amid the rising numbers of uninsured and underinsured people and declining resources. Despite this, there remains a limited understanding of what research and funding should be prioritized given the needs of those most burdened and impacted by disparities, and CHCs serving these communities. The objective of this study was to identify and prioritize health equity-focused research topics among key advisors within CHCs and their communities.

## Methods

The University of California San Francisco (UCSF) Clinical and Translational Science Institute [13] (CTSI) community engagement program aims to increase community participation in all stages of research by bridging academic research, health policy and community practice to improve public health. This includes a practice-based research network, called the San Francisco Bay Area Collaborative Research Network [14] (SFBayCRN) designed to encourage, facilitate, and lead mutually beneficial research partnerships between researchers, CHCs and the community. With guidance and support from the CTSI and the SFBayCRN, we launched the Health Equity covid-19 REsearch with CHCs (HERE with Community) project in 2021, to identify infrastructure and resources needed and recommendations to support partnerships between CHCs, academic medical centers, and the community, to enable advisor-engaged health equity-focused research. This study was approved by the UCSF Institutional Review Board (IRB#21-35225) as exempt under category 2: research that only includes interactions involving educational tests, survey procedures, interview procedures, or observation of public behavior. Two well-established, validated methods guided our collaborative, inclusive, and consultative approach to community engagement for health equity-focused research topic prioritization to ensure the prioritized research topics are driven by the needs and priorities of those most impacted:

1. The Patient-Centered Outcomes Research Institute (PCORI) standards for formulating patient-centered research topics, which includes methods for community engagement that ensure the representativeness of engaged groups and dissemination of study results [15]. PCORI is an independent, nonprofit research funding organization that seeks to empower patients and others with actionable information about their health and healthcare choices, by funding research that helps people make better-informed healthcare decisions based on their needs and preferences [16].
2. The James Lind Alliance (JLA) approach to “priority setting partnerships” through which community members, clinicians, and researchers partner to identify and prioritize unanswered questions, with core principles of inclusivity, collaboration, transparency and focus on evidence [17].

**Figure 1.**
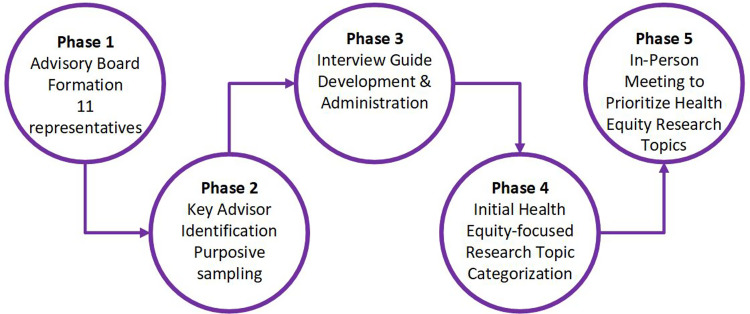
Summary of HERE with Community methods to identify and prioritize health equity-focused research topics.

HERE with Community used qualitative methods, including data triangulation across five stepwise phases to build a more robust and credible understanding of prioritizes for health equity-focused research topics within CHCs and their key advisors. The phases include community advisory board engagement (Phases 1 and 2), individual and small group interviews (Phase 3), categorization of interview findings with our advisory board (Phase 4), and nominal group technique prioritization (Phase 5). Our process is described in detail below and summarized in Figure 1. This study follows the Consolidated Criteria for Reporting Qualitative Research (COREQ) guidelines to ensure consistency, quality and rigor [18].

### Phase 1: Community advisory board formation

We created a community advisory board comprised of 11 diverse representatives from CHC partnering networks (see Table 1), including Benioff Children’s Hospital (BCH) Oakland, Family HealthCare Network (FHCN) in the Central Valley of California, San Francisco Community Clinic Consortium (SFCCC), as well as the UCSF and UCSF Fresno. The advisory board included 5 CHC community members, 4 CHC clinicians/leaders and 2 researchers, and overall consisted of 5 men, 6 women, and 7 people of color. CHC community members were identified from local CHC advisory boards from partnering community networks. We met every two months for a total of 12 meetings throughout the project (median attendance: 9, range: 7–9). All advisory board members were compensated $125 per hour throughout the project in recognition of their time, expertise, and participation. Advisory board members participated in all research phases (see below) – including identification of key informants, finalization of the interview guide, data analysis, emerging results, and interpretation, finalizing research topic prioritization; reviewed and provided feedback on manuscript drafts, and approved the final version prior to submission and are included as co-authors (see Author Affiliations).

**Table 1.** Summary and description of partnering community health center networks

**Benioff Children’s Hospital (BCH) Oakland Federally Qualified Health Centers** BCH Oakland provides care to ethnically diverse children and adolescents living in low-income neighborhoods in Alameda County, California. BCH Oakland has relationships with over 20 community organizations serving children and families outside the traditional healthcare system and developed special programs for diverse patient populations. One clinician and one community member/patient advisor participated in the CAB.
**Family Healthcare Network (FHCN) in the Central Valley of California** The FHCN is a nonprofit community-based organization that operates 41 sites, 36 of which are Federally Qualified Health Centers in Tulare, Kings, and Fresno Counties in California’s Central Valley. These centers are one of the largest providers of care for medically underserved populations in the United States. One clinician and one community member/patient advisor participated in the CAB.
**San Francisco Community Clinic Consortium (SFCCC**) The SFCCC is a consortium of 12 non-profit CHCs with 27 sites providing care to San Francisco’s low-income, uninsured, and medically underserved people. Many of the SFCCC CHCs emphasize services to populations with special needs, such as racial and ethnic minorities, sexual minorities, immigrants, and people experiencing homelessness. One clinician and two community members/patient advisors participated in the CAB.
**University of California San Francisco (UCSF) Clinical & Translational Science Institute** UCSF is the leading university exclusively focused on health through advanced biomedical research, graduate-level education in the life sciences and health professions, and excellence in patient care. Two researchers with community engagement experience participated in the CAB.
**UCSF Fresno** UCSF Fresno is a major clinical and educational branch of UCSF. UCSF Fresno serves as a hub for medical education and research in the San Joaquin Valley and Central California. One clinician and one community member participated in the CAB

### Phase 2: Key informant identification

We used purposive, criterion-i sampling [19] to invite key informants connected to our CHC network partners to participate in individual or small groups interviews via Zoom, based on availability, to yield the most useful information given our study objective. Our advisory board created a list of key informants who met one or more of the following criteria:

- Individuals who were leaders, clinicians, staff, or patients from the SFCCC, BCH Oakland or FHCN.
- Individuals who were leaders, clinicians, researchers from UCSF, UCSF BCH Oakland, or UCSF Fresno with previous experience conducting practice-based and/or community-engaged research in CHC settings.
- Leaders of community organizations from the Central Valley of California.
- Members of the farm working communities from the Central Valley of California.

Potential participants identified by our advisory board members were sent an invitation email that invited them to take part in the study.

### Phase 3: Individual/Small group interview guide development and administration

We developed a study-specific interview guide in partnership with our community advisory board members to explore participants’ experience and identify infrastructure and resources needed to support priorities for health equity research between academic researchers, CHCs, and community members, related to patient-centered outcomes research. Draft questions were reviewed and revised accordingly, keeping in mind different audiences (e.g., researchers, CHC staff, and patient/community members) based on feedback and discussion with the advisory board. Interviews started with an introduction of the facilitators and their positionality in context of the research (NRP and JDH), the study purpose, what we hope to learn from participants’ experiences, definitions of patient-centered outcomes research and comparative effectiveness research, instructions and guidelines, and then opened with introductions from participants, followed by interview questions. Interview questions explored respondents’ experience with COVID-19, community-engaged research, and how community members, CHC networks and academic institutions can partner to achieve health equity, including research priorities for their community (see Supplementary Material 1). Participants self-selected to participate in either an individual or small group interview based on their preference and availability. All individual and small group interviews (2–3 participants) were scheduled for one-hour and conducted via Zoom by team members (NRP and JDH), who have extensive training and experience in qualitative research methods and facilitation. All participants received a $125 gift card for their participation. All interview sessions were digitally recorded and professionally transcribed verbatim. We also asked our advisory board members to share their perspective on health equity priorities for their communities during one of our bi-monthly advisory board meetings.

### Phase 4: Initial health equity-focused research topic categorization

Several steps were taken to ensure theoretical and methodological rigor and credibility throughout the analysis. Two members of the project study team (NRP and JDH) independently performed qualitative content analysis to categorize all suggested research topics [20,21]. This analytic approach identifies, analyzes, and reports patterns within the data. We used to an inductive (data-driven) approach to coding. This included sustained immersion and familiarization with the data, ensuring multivocality, reflexive memoing, independent coding, then group reviewing to reach coding consensus, maintenance of an electronic paper trail of analytic decisions, and frequent research team meetings to review code and theme development [22–24]. All coding disparities were discussed and resolved by negotiated consensus. During the analysis process, the study team met bi-monthly with the advisory board to discuss the analysis process and emerging results to get iterative feedback to finalize research topic categorization.

### Phase 5: In-person meeting for final topic prioritization

We compiled a full list of topics identified from content analysis and presented them to attendees of a break-out session at the SFBayCRN Annual Meeting. Attendees typically represent patients, caregivers, community members, clinicians, researchers, and leaders of various community organizations. We did not provide attendees details on how many times each topic was discussed by interview participants or the advisory board, as this could potentially bias attendees’ decision-making process, in which their original perspectives would not be shared.

We then used nominal group technique (NGT) – a prioritization methodology – that facilitates inclusiveness and ensures a consensus-driven process during the research topic prioritization processes [25]. NGT was chosen to allow all voices and perspectives, including quieter individuals, to be heard by removing power dynamics and enables effective group decision-making and ownership among advisors. It also prevents groupthink phenomenon where conformity (social desirability bias) override critical individual thinking and expression of differing opinions. The NGT process commenced by meeting attendees being randomly assigned to one of six smaller groups. Each group had a facilitator and a notetaker who were either UCSF staff, representatives of a CHC network partner, or member of our advisory board. The NGT process included the following steps:

- Starting with the question, “*What COVID-19-related health equity research topics are important for your community?*,” within small groups, each participant shared their ideas without discussion until all participants had an opportunity to share (i.e., silent generation of ideas in round-robin style).
- This was then followed by an open discussion, in which participants could ask clarifying questions, elaborate on responses, combine or split ideas, or add new ones.
- Facilitators then compiled a full list of topics and question identified, with assistance from the notetaker.
- Each participant then quietly ranked their top 3 topics/questions and then shared them within the group.
- Each small group was then asked to come up with their collective top 3 to 5 topics/questions that was shared during the larger group discussion, with live notetaking.
- As a full group (all small groups combined), a final list of research topics/questions was created by consensus and ranked the top five topics across all groups.

The small and large group discussions were digitally recorded and transcribed. We also had a live notetaker for the full/larger group discussion, that made it possible to present the final top five prioritized health equity research topics at the end of our session.

### Triangulation

Study findings were triangulated using three different methods across the five study Phases: individual and small group interviews (Phase 3), iterative feedback on qualitative analysis from advisory board (Phase 4), and NGT prioritization results (Phase 5). These three approaches allow insights that can reveal dissimilar views from a variety of people on the topic under investigation – i.e., health equity research priorities [26]. Investigator triangulation was also implemented to avoid potential bias with the integration of (1) individual reviewing, coding and memoing by two researchers (NRP and JDH), (2) consensus discussions between the two researchers, and (3) iterative feedback with the research team and advisory board. These two triangulation strategies increase the rigor, credibility, and trustworthiness of the data collected and study findings [26], which adds breadth to our understanding of health equity research priorities.

## Results

### Participants

Forty-eight people received emailed invitation to take part in the study. Of the 48 invitees, 4 researchers did not participant, and the remaining 44 participants scheduled and completed interviews (92% response rate). Most of the participants were female (61%) and people of color (80%). Thirteen individual interviews and 7 small group interviews were completed with 44 participants from the SFCCC (*n* = 6), BCH Oakland (*n* = 4), FHCN (*n* = 5), UCSF (*n* = 8) and UCSF Fresno (*n* = 2), and Central Valley farmworkers and community leaders (*n* = 19). Key advisors represented CHC staff and leaders (27%), researchers (27%), and community organization leaders or community members (46%) (see Table 2).

**Table 2.** Individual/small group interview participants by type of advisor

Group represented	Number of individual interviews	Small group interviews		
		# of groups	# of participants	N
**Researchers**				**12**
BCH Oakland	2	–	–	2
UCSF	–	3	2, 3, 3	8
UCSF Fresno	–	1	2	2
**Community health centers/clinicians**				**12**
FHCN	3	1	2	5
SFCCC	6	–	–	6
BCH Oakland	1	–	–	1
**Patients/community members and leaders**				**20**
BCH Oakland	1	–	–	1
Central Valley community organizations	–	1	8	8
Central Valley farmworkers	–	1	11	11
**TOTAL**	**13**	[7 groups]	**31**	**44**

BCH Oakland = Benioff Children’s Hospital Oakland; FHCN = Family HealthCare Network; SFCCC = San Francisco Community Clinic Consortium; UCSF = University of California San Francisco.

### Individual/small group interviews and advisory board

A total of 11 Initial health equity-focused research themes identified from our interviews and advisory board meetings are outlined below and summarized in Table 3. Initial topics related to COVID-19 and impact from the pandemic (e.g, vaccine hesitancy, psychological impact on children and youth, and long-term side effects from COVID), along with non-COVID-related topics that illustrate prevalent disparities in our healthcare system that were exacerbated during the pandemic (e.g., mental health crisis and lack of resources, access to and delivery of healthcare services, and addressing misinformation and building trust in healthcare). Representative quotes from study participants related to these research themes are listed in Supplementary Material 2. Details of all research topics identified from individual and small group interviews and advisory board meetings are listed in Supplementary Material 3.

**Table 3.** Initial unprioritized health equity-focused topic categorization identified during focus groups, interviews, and advisory board meetings

1) Vaccines: hesitancy, barriers, misinformation, messaging fatigue* 2) Mental & Behavioral Health: mental health crisis, overall impact* 3) Children & Youth: psychological and educational impact* 4) Translational Research: biological reasons for variation in immune response 5) CHCs’ Workforce: shortage, burnout, sustainable investment* 6) Long COVID: overall, mental health, farmworkers, essential workers, Latino/a* 7) Healthcare Delivery: optimizing telehealth, managing backlogs of preventive / chronic conditions, sustaining CHC service model, improving next pandemic response 8) Outcomes & Disparities: overall descriptions, essential workers, diverse populations, social determinants of health, interaction with other chronic conditions, access to vaccines and effectiveness, access to and outcomes of paxlovid/antivirals* 9) Trust in healthcare & science: addressing misinformation* 10) Research methods: CHC participation in research trials and access to results 11) Anti-racism: anti-Asian hate because of COVID

* Topics highlighted with an asterisk were noted by multiple advisors.

### Final consensus and prioritization of research topics

Ninety participants took part in our breakout session at the SFBayCRN annual meeting. Based on affiliation noted in meeting registration, participants included approximately 25 patients, caregivers, and community members and leaders (28%); approximately 35 participants were clinicians (39%); and approximately 30 participants were researchers or organizational leaders (33%). Attendees to this meeting included members of the SFBayCRN, members of our study advisory board, community members, representatives from patient and family advisory councils and community advisory boards for clinics and community-based organizations, academic researchers, primary care providers, and clinicians from California who represent diverse communities. Following the use of NGT, six overarching themes were identified and several sub-themes and questions relating to mental health challenges among specific subpopulations and the impact of isolation and loss, improving trust in healthcare and science, healthcare delivery models to improve access for underrepresented populations, building a healthcare system and conducting research through an anti-racism lens, addressing misinformation in healthcare and specific subpopulations, and sustaining lessons learned from policies that improved access to care during the pandemic. Topics are listed in rank order of priority (see Table 4). Other research topics noted during group discussions included long COVID, vaccine hesitancy, telehealth access, access to health care technology for immigrant communities, how to ethically engage underrepresented communities, and lessons from the positive/negative impacts of COVID.

**Table 4.** Prioritized health equity focused research topics

**1. Mental health** Specific populations noted are children, seniors, primary care providers and the overall healthcare workforce. The impact of isolation and loss on mental health.
**2. Trust** Strategies and interventions to improve the public’s trust in healthcare and science.
**3. Healthcare delivery** Healthcare delivery models that increase access to healthcare for underrepresented communities (general healthcare access). Strategies and interventions to address the backlog of health care in the community. Building an anti-racist healthcare system. Therapeutic alliance – changing how we practice healthcare, time allotted, earning trust and building relationships with patients, and primary care needed to fill gaps due to scarcity of mental health providers.
**4. Anti-racism** View all health care issues through an anti-racism lens. Building an anti-racist healthcare system that assesses outcomes/health disparities. Ensure access to all health care services for those with limited English proficiency
**5. Misinformation – strategies and interventions** Specific populations noted: immigrant populations. Increase trust and transparency of healthcare information Address messaging fatigue
**6. Institutionalize lessons learned** Continuation/discontinuation of COVID policies (Medicare, Medicaid, policy)

## Discussion

Using a dynamic and collaborative engagement process, we identified 6 prioritized topics for health equity research, including: addressing mental health challenges, improving trust in healthcare and science, healthcare delivery models to improve access and utilization, building and sustaining an anti-racism lens in healthcare access, strategies and interventions to address misinformation, and institutionalizing lessons learned from the pandemic. While some of the topics are well-known and persistent topics in need of research, our findings highlight community informed challenges within our healthcare system that merit attention, particularly as the healthcare landscape continues to evolve.

Mental health challenges were identified as a top priority. This is not surprising given the increasing rates of psychological distress in the US, particularly among subpopulations such as children and young adults who have experienced larger deteriorations in mental health than their older counterparts [27]. People who are medically underserved and socially marginalized populations are also greatly impacted by mental health challenges given the “triple pandemic” of exacerbated disparities and inequities in health, justice, and economics [28]. Despite the prevalence of mental health issues, approximately 60% of people with any mental health condition never receive treatment due to barriers to care (e.g., insurance, stigma, resource-strained healthcare workforce, etc.) [29]. As mental health needs persist, current responses are insufficient and inadequate [30], and research that addresses barriers to access effective mental health services must be prioritized. The cross collaboration of varied community and healthcare advisors are needed to reshape the environments that influence mental health and strengthen mental health care systems.

Identifying strategies and interventions to improve the public’s trust in healthcare and science is a notable key priority. Respondents highlighted a lack of trust between patients, communities, healthcare systems, and science. This concept is not new as over the past half century, trust in the US healthcare system has consistently declined [31], along with public trust in the federal government [32]. Trust is critical to develop and maintain the health and wellbeing of individuals, communities, and societies. Historically, public health practitioners assume patients and the public will trust them because of their training and position in society [33]. However, trust must be earned and sustained and built through relational community partnerships. Building and maintaining trust in public health institutions is crucial for meeting the needs of patients and the community, and to ensure positive work environments for all employees (e.g., healthcare workers) [33]. Notably, lay health workers (e.g., promotoras) are known as community trust builders in public health and contributed greatly to promoting health during the pandemic [34], along with building relational partnerships within the community.

Our findings highlighted the need to combat health misinformation, as it impacts health behaviors – e.g., noncompliance with public health recommendations [35]. The decline of trust and confidence in healthcare, government and news media is associated with the rapid spread of inaccurate information [36], and merits development of strategies and interventions to address misinformation. A recent study showed trust in different types of media (broadcast, print, and social) varied by age, race and ethnicity, income level, and political party affiliation, and contributed to the adoption of preventive behaviors [37] and should be considered for intervention development. Understanding, managing, and intervening on the sources and spread of misinformation in public health and engaging trusted information sources is critical to achieve heath equity. Furthermore, many people have low science literacy – the ability to make well-informed decisions based on facts, research, and knowledge, not on opinion or hearsay – as it can be difficult to discern between what is fake and what is real [38]. Trusted community partnerships can help increase knowledge and understanding of science and health information to help people make well-informed decisions.

Issues with access to and utilization of healthcare services also emerged as a priority to achieve health equity. Access to healthcare remains challenging for low-income under/uninsured patients, people with undocumented status, and those in living in remote areas [39]. CHCs provide primary care and other services to medically underserved patients; however, there has been a reduction in use of health services and exacerbation of barriers to care [40]. For example, barriers to access specialty care among CHC patients persists, despite expansion of Medicaid [41]. Investments in CHCs and expansion of public insurance programs can improve access to care and breadth of services available, as CHCs and community health workers are integral to expand access to services and require robust infrastructure for growth [39]. And while one resource, the use of telemedicine, facilitated access to healthcare in remote areas and during the pandemic with the relaxation of federal government regulatory barriers, challenges persist among under-resourced communities that lack access to basic technologies necessary for successful deployment and use [42]. Investments in broadband access, equipment and education can reduce the digital divide and increase use of and comfort with technologies [42]. Overall, it is critical for research to address the individual, structural, and systemic determinants of healthcare access and utilization.

Associated with improving healthcare access and delivery is the identified priority to build an anti-racist healthcare system, which is consistent with research on health equity [43,44]. Racism is a structural determinant of heath inequities that is rarely taught, cited, or targeted as the root cause of inequities [45]. Building an anti-racist healthcare system starts with acknowledging racial biases, heighten racial consciousness, and name and identify racism to challenge it [44]. Within this context, medical education can address historical roots of scientific exploitation, implicit bias, and institutionalized practices and policies that impact clinical interactions and patients’ lives [45,46]. Contemporary studies have also outlined processes, principles, and strategies to implement anti-racism interventions within healthcare settings that merit further exploration [47].

Our results highlight future research should also examine the lessons learned from the pandemic that merit expansion or permanent institutionalization to achieve health equity. For example, access to care was greatly expanded during the pandemic due to regulatory flexibility that supported providers’ financial sustainability – including widespread adoption of telehealth [48]. Academic medical institutions responded to the behavioral health needs of the healthcare workforce with mental health and emotional well-being programs given the growing mental health crisis of increased burnout, depression, anxiety and stress [49]. Study respondents also noted the necessity of broad community partnerships and engagement of key community advisors that had positive impacts on access to healthcare for underserved communities, which is reflected in the literature [50].

This study was strengthened by the diverse perspectives and experiences of patients and communities most burdened by disparities, the CHCs that care for them, and academic researchers committed to health equity. Additionally, healthy equity topics were systematically identified using rigors qualitative methods, including method triangulation across study phases and investigator triangulation for analysis. However, there are notable limitations. First, the opinions of our interview participants may not be representative of all diverse perspectives of CHC advisors, patients/community members, and academic researchers, as we focused on California, and we did not collect sociodemographic data to confirm diversity. Second, our CHC partnerships were limited to those with whom we had working relationships and may not represent a broad range of CHCs and community members. Similarly, our advisors’ perspectives may not be completely representative of the organizations, as there may be relevant groups who were not represented in our sample.

### Next steps and future directions

Findings from this work identified gaps in healthcare, workplace protections, and education for farmworkers and their families, which demonstrate a strong need for advocacy for healthcare, social and financial policy changes within CHCs, academic centers, and communities in the Central Valley. This work further supports prioritizing active listening and collaborative engagement with diverse community partners, beyond academic researchers, to address topics truly meaningful to underserved communities and effectively reduce health disparities [7]. As a result, our team presented these findings to institutional and research leaders across partnering organizations to increase awareness and encourage change through the creation of new strategic priorities. We also secured additional funds to create a video to disseminate our findings and policy implications related to the needs of farmworkers and community organizations in the Central Valley. The target audience for this video will be local and state-based politicians and representatives who can use this work to advocate for new funding, inform the creation of bills for farmworker protection and healthcare access, and inform new strategic priorities. We also expect the video will be used by community organizations to help them further advocate for ongoing resources. We continue to engage our advisory board and their networks through completion of our dissemination plans and development of future community-engaged initiatives.

## Conclusion

Conducting research across these areas presents various challenges, as trusted relationships, infrastructure, and research funding are all required to move these agendas forward with input from key community informants. Although numerous studies have reported on the infrastructure required to conduct research in CHCs and federally qualified health centers [6,51,52], few studies have completed community-engaged prioritization to identify notable research topics that align with CHC priorities [53–55]. It is also well-known that research topics often do not align with CHC priorities and patient/community needs [53–55]. This community-engaged study highlights the topics that should be prioritized in future research that will necessitate sustainable infrastructure and funding to move these agendas forward. Several institutional and national initiatives can also play a role in academic-community partnerships translating prioritized research topics into action. For example, Clinical and Translational Science Awards (CTSAs) within academic institutions have an opportunity to facilitate high-level discussions and academic-community partnership agreements [12]; and Practice-Based Research Network (PBRN) can have an impact at the local and national level to enhance research [56].

In summary, centering the voices and experiences of key advisors within CHCs and the communities they serve is powerful, enlightening, and essential to achieve health equity. Overall, our community advisors highlighted needs related to health equity and persistent disparities that merit future research, including: addressing mental health challenges, improving trust, developing strategies and interventions to address misinformation, improving healthcare access and utilization, ensuring anti-racist healthcare delivery, and institutionalizing lessons learned from the pandemic that should be maintained to enhance healthcare access for medically underserved patients. Community advisors are essential to moving these research agendas forward, identifying potential solutions, and subsequently should be at future research planning tables and engaged in research and advisory boards across academic institutions and CHCs. Our findings offer important directions to advance health equity-focused research to meet the needs of medically underserved communities burdened by persistent health disparities.

## Supporting information

10.1017/cts.2025.10179.sm001 Palmer et al. supplementary material Palmer et al. supplementary material

## Supplementary material

The supplementary material for this article can be found at https://doi.org/10.1017/cts.2025.10179.

## References

[1] Beeson T , Jester M , Proser M , Shin P. Engaging community health centers (CHCs) in research partnerships: The role of prior research experience on perceived needs and challenges. Clin Transl Sci. 2014;7:115–120. doi: 10.1111/cts.12150. 24774327 PMC5350950

[2] Gyamfi J , Peprah E. Scaling-up evidence-based interventions for communities of color with marked health disparities: Lessons learned from COVID-19 can be applied to reduce morbidity and mortality and achieve health equity. Med Care. 2023;61:417–420. doi: 10.1097/MLR.0000000000001872. 37289562

[3] Wallerstein N , Oetzel JG , Sanchez-Youngman S , et al. Engage for equity: A long-term study of community-based participatory research and community-engaged research practices and outcomes. Health Educ Behav. 2020;47:380–390. doi: 10.1177/1090198119897075. 32437293 PMC8093095

[4] National Association of Community Health Centers. What is a community health center? (https://www.nachc.org/about/about-our-health-centers/what-is-a-health-center/) Accessed May 16, 2021.

[5] Hébert JR , Adams SA , Ureda JR , et al. Accelerating research collaborations between academia and federally qualified health centers: Suggestions shaped by history. Public Health Rep. 2017;133:22–28. doi: 10.1177/0033354917742127. 29182891 PMC5805099

[6] Brandt HM , Young VM , Campbell DA , Choi SK , Seel JS , Friedman DB. Federally qualified health centers’ capacity and readiness for research collaborations: Implications for clinical-academic-community partnerships. Clin Transl Sci. 2015;8:391–393. doi: 10.1111/cts.12272. 25962873 PMC4553115

[7] D’Agostino EM , Oto-Kent D , Nuño M. Paving the way for the next frontier of community-engaged research. Am J Public Health. 2024;114:S347–S349. 38776499 10.2105/AJPH.2024.307685 PMC11111370

[8] Irby MB , Moore KR , Mann-Jackson L , et al. Community-engaged research: Common themes and needs identified by investigators and research teams at an emerging academic learning health system. Int J Env Res Public Health. 2021;18:3893. doi: 10.3390/ijerph18083893. 33917675 PMC8068003

[9] Jung OS , Begum F , Dorbu A , et al. Ideas from the frontline: Improvement opportunities in federally qualified health centers. J Gen Intern Med. 2023;38:2888–2897. doi: 10.1007/s11606-023-08294-1. 37460922 PMC10593646

[10] Chin MH , Kirchhoff AC , Schlotthauer AE , et al. Sustaining quality improvement in community health centers: Perceptions of leaders and staff. J Ambul Care Manage. 2008;31:319–329. doi: 10.1097/01.JAC.0000336551.67922.2f. 18806592 PMC2659650

[11] LeBlanc M , Radix A , Sava L , et al. Focus more on what’s right instead of what’s wrong: ‘Research priorities identified by a sample of transgender and gender diverse community health center patients. BMC Public Health. 2022;22:1741. doi: 10.1186/s12889-022-14139-z. 36104812 PMC9472366

[12] Towfighi A , Orechwa AZ , Aragón TJ , et al. Bridging the gap between research, policy, and practice: Lessons learned from academic – public partnerships in the CTSA network. J Clin Transl Sci. 2020;4:201–208. 32695489 10.1017/cts.2020.23 PMC7348007

[13] The University of California San Francisco. Clinical and translational science institute. (https://ctsi.ucsf.edu/) Accessed December 18, 2023.

[14] The University of California San Francisco. Clinical and translational science institute. San Francisco Bay Collaborative Research Network. (https://consult.ucsf.edu/sfbaycrn) Accessed December 18, 2023.

[15] Patient-Centered Outcomes Research Institute (PCORI). PCORI methodology standards: Standards for formulating research questions. (https://www.pcori.org/research/about-our-research/research-methodology/pcori-methodology-standards#Formulating%20Research%20Questions) Accessed December 18, 2023.

[16] Patient-Centered Outcomes Research Institute (PCORI). About PCORI. (https://www.pcori.org/about/about-pcori) Accessed October 24, 2024.

[17] Alliance James Lind. The James Lind Alliance Guidebook. Version 8. Southampton: James Lind Alliance, 2018.

[18] Tong A , Sainsbury P , Craig J. Consolidated criteria for reporting qualitative research (COREQ): A 32-item checklist for interviews and focus groups. Int J Qual Health Care. 2007;19:349–357. doi: 10.1093/intqhc/mzm042. 17872937

[19] Palinkas LA , Horwitz SM , Green CA , Wisdom JP , Duan N , Hoagwood K. Purposeful sampling for qualitative data collection and analysis in mixed method implementation research. Adm Policy Ment Health. 2015;42:533–544. doi: 10.1007/s10488-013-0528-y. 24193818 PMC4012002

[20] Schreier M. Qualitative Content Analysis in Practice. Los Angeles: Sage Publications, 2012.

[21] Bradley EH , Curry LA , Devers KJ. Qualitative data analysis for health services research: Developing taxonomy, themes and theory. Health Serv Res. 2007;42:1758–1772. 17286625 10.1111/j.1475-6773.2006.00684.x PMC1955280

[22] Tracy SJ. Qualitative quality: Eight “big-tent” criteria for excellent qualitative research. Qual Inq. 2010;16:837–851. doi: 10.1177/1077800410383121.

[23] Whittemore R , Chase SK , Mandle CL. Validity in qualitative research. Qual Health Res. 2001;11:522–537. doi: 10.1177/104973201129119299. 11521609

[24] Morse JM. Critical analysis of strategies for determining rigor in qualitative inquiry. Qual Health Res. 2015;25:1212–1222. doi: 10.1177/1049732315588501. 26184336

[25] Centers for Disease Control and Prevention. *Evaluation briefs: Gaining Consensus Among Stakeholders Through the Nominal Group Technique*. Evaluation Research Team. U.S. Department of Health and Human Services. Centers for Disease Control and Prevention. Atlanta, GA, 2018. (https://www.cdc.gov/healthyyouth/evaluation/pdf/brief7.pdf) Accessed December 18, 2023.

[26] Morgan H. Using triangulation and crystallization to make qualitative studies trustworthy and rigorous. The Qualitative Report. 2024;29:1844–1856.

[27] Ruhm CJ. Mental health and mortality trends in the United States. J Health Econ. 2025;102:103015. doi: 10.1016/j.jhealeco.2025.103015. 40466290

[28] Aditya T , Kosasih A , Puspita DR. Racial equity, COVID-19, and public policy: The triple pandemic (1st Edition): by Harper-Anderson EL, Albanese JS, Gooden ST, Routlegde, New York. Crit Policy Stud. 2023;18(1):160–162. doi: 10.1080/19460171.2023.2268678.

[29] Ward-Ciesielski EF , Rizvi SL. Finding mental health providers in the United States: A national survey and implications for policy and practice. J Ment Health. 2019;30:578–584. doi: 10.1080/09638237.2019.1677867. 31647364

[30] Freeman M. The world mental health report: Transforming mental health for all. World Psychiatry. 2022;21:391–392. doi: 10.1002/wps.21018. 36073688 PMC9453907

[31] Khullar D. Building trust in health care: Why, where, and how. JAMA. 2019;322:507–509. doi: 10.1001/jama.2019.4892. 31305868

[32] Public Trust in Government: 1958–2023. Pew research center. 2023. (https://www.pewresearch.org/politics/2023/09/19/public-trust-in-government-1958-2023/#:) Accessed December 18, 2023.

[33] Ward PR. Improving access to, use of, and outcomes from public health programs: The importance of building and maintaining trust with patients/Clients. Front Public Health. 2017;5:22. doi: 10.3389/fpubh.2017.00022. 28337430 PMC5340761

[34] Rämgård M , Ramji R , Kottorp A , Forss KS. ‘No one size fits all’ – Community trust-building as a strategy to reduce COVID-19-related health disparities. BMC Public Health. 2023;23:18. doi: 10.1186/s12889-022-14936-6. 36597039 PMC9810513

[35] Jin SL , Kolis J , Parker J , et al. Social histories of public health misinformation and infodemics: Case studies of four pandemics. Lancet Infect Dis. 2024;24:e638–e646. doi: 10.1016/S1473-3099(24)00105-1. 38648811

[36] Schluter AP , Généreux M , Landaverde E , Schluter PJ. In the COVID-19 pandemic, who did we trust? An eight-country cross-sectional study. J Glob Health. 2023;13:06036. doi: 10.7189/jogh.13.06036. 37651637 PMC10471152

[37] Li H , Chen B , Chen Z , et al. Trust in COVID-19 information from different media types and its association with preventive measures adoption in the U.S. J Health Commun. 2023;28:633–647. doi: 10.1080/10810730.2023.2245373. 37665096 PMC11929583

[38] Cammack S , Boehm MJ , Lodl K , et al. Science literacy: Using research-based facts to make real-world decisions. 2018. (https://digitalcommons.unl.edu/cgi/viewcontent.cgi?article=1012&context=sdn) Accessed December 18, 2023.

[39] Saloner B , Wilk AS , Levin J. Community health centers and access to care among underserved populations: A synthesis review. Med Care Res Rev. 2020;77:3–18. doi: 10.1177/1077558719848283. 31079529

[40] Pujolar G , Oliver-Anglès A , Vargas I , Vázquez M-L. Changes in access to health services during the COVID-19 pandemic: A scoping review. Int J Environ Res Public Health. 2022;19:1749. doi: 10.3390/ijerph19031749. 35162772 PMC8834942

[41] Timbie JW , Kranz AM , Mahmud A , Damberg CL. Specialty care access for medicaid enrollees in expansion states. Am J Manag Care. 2019;25:e83–e87. 30875176 PMC6986199

[42] Gallegos-Rejas VM , Thomas EE , Kelly JT , Smith AC. A multi-stakeholder approach is needed to reduce the digital divide and encourage equitable access to telehealth. J Telemed Telecare. 2023;29:73–78. doi: 10.1177/1357633X221107995. 35733379

[43] Beech BM , Ford C , Thorpe RJ Jr , Bruce MA , Norris KC. Poverty, racism, and the public health crisis in America. Front Public Health. 2021;9:699049. doi: 10.3389/fpubh.2021.699049. 34552904 PMC8450438

[44] Bailey ZD , Krieger N , Agénor M , Graves J , Linos N , Bassett MT. Structural racism and health inequities in the USA: Evidence and interventions. Lancet. 2017;389:1453–1463. doi: 10.1016/S0140-6736(17)30569-X. 28402827

[45] Mabeza RM , Legha RK. Reimagining medical education toward antiracist praxis. Health Equity. 2023;7:598–602. doi: 10.1089/heq.2023.0135. 37731791 PMC10507935

[46] Wilkins CH , Williams M , Kaur K , DeBaun MR. Academic medicine’s journey toward racial equity must be grounded in history: Recommendations for becoming an antiracist academic medical center. Acad Med. 2021;96:1507–1512. doi: 10.1097/ACM.0000000000004374. 34432719 PMC8542070

[47] Hassen N , Lofters A , Michael S , Mall A , Pinto AD , Rackal J. Implementing anti-racism interventions in healthcare settings: A scoping review. Int J Environ Res Public Health. 2021;18:2993. doi: 10.3390/ijerph18062993. 33803942 PMC8000324

[48] Chaudhry HJ. Expanding licensure portability and access to care: Lessons learned during COVID-19. Health Aff (Millwood). 2022;41:1136–1138. doi: 10.1377/hlthaff.2022.00756. 35914196

[49] Mangurian C , Fitelson E , Devlin M , et al. Envisioning the future of well-being efforts for health care workers-successes and lessons learned from the COVID-19 pandemic. Jama Psychiat. 2023;80:962–967. doi: 10.1001/jamapsychiatry.2023.2355. 37494012

[50] Malika N , Herman PM , Whitley M , et al. Qualitative assessment CIH institutions engagement with underserved communities to enhance healthcare access and utilization. Glob adv integr med health. Glob Adv Integr Med Health. 2024;13:27536130241244759. doi: 10.1177/27536130241244759. 38545335 PMC10966973

[51] Likumahuwa S , Song H , Singal R , et al. Building research infrastructure in community health centers: A community health applied research network (CHARN) report. J Am Board Fam Med. 2013;26:579–587. doi: 10.3122/jabfm.2013.05.130025. 24004710 PMC4559143

[52] Walter E , O’brien M. Research infrastructure and capacity in federally qualified health centers. J Health Care Poor Underserved. 2025;36:410–415. doi: 10.1353/hpu.2025.a951607. 39957660 PMC12147091

[53] Crowe S , Fenton M , Hall M , Cowan K , Chalmers I. Patients, clinicians and the research communities’ priorities for treatment research: There is an important mismatch. Res Involv Engagem. 2015;1:2. doi: 10.1186/s40900-015-0014-7. Erratum in: *Res Involv Engagem*. 2015 Dec 23;**1**:14. 29062491 PMC5598091

[54] Stewart R , Oliver S. A Systematic Map of Studies of Patients’ and Clinicians’ Research Priorities. London: James Lind Alliance, 2008.

[55] Manafò E , Petermann L , Vandall-Walker V , Mason-Lai P. Patient and public engagement in priority setting: A systematic rapid review of the literature. Plos One. 2018;13:e0193579. doi: 10.1371/journal.pone.0193579. 29499043 PMC5834195

[56] Agency for Healthcare Research & Quality. Primary care practice-based research networks. 2018. (https://www.ahrq.gov/research/findings/factsheets/primary/pbrn/index.html) Accessed July 15, 2025.

