# Supplementary material for: Engaging community health center advisors to identify research priorities for health equity
Source: J Clin Transl Sci. 2025 Oct 28;9(1):e253. doi: 10.1017/cts.2025.10179 (PMC12766519; doi:10.1017/cts.2025.10179)
Supplement: Palmer et al. supplementary material [file S2059866125101799sup001.docx]

**Supplementary Material 1**

**Interview Guide**

***HERE with Community***

Health Equity covid-19 REsearch with Community health centers

Advisory Groups:

- Clinic leadership (English)
- Clinic staff (English)
- Patients/community advisory board members (English & Spanish)
- Academic researchers (English)

***INTRODUCTION***

Welcome! First, we want to thank all of you for taking the time to participate in this focus group discussion. I am _(facilitator)_. I will be the facilitator of this discussion group. This is _(recorder/note taker)_. S/he will take notes while we talk.

The purpose of this meeting is to identify the infrastructure and resources needed to support priorities for health equity focused COVID-19 PCOR/CER between academic researchers and community health centers, such as Federally Qualified Health Centers (FQHCs). This will include identifying community assets and strengths. We want to learn about what things are important to you, your patient and/or community partners to address COVID-19. We want to learn from your experiences, so that we can identify strategies to strengthen community health center partnerships with academic researchers. By talking about your experiences and what is important to your community, we hope to better understand how patients, communities, and universities work together in order to conduct health equity focused COVID-19 PCOR/CER. We are interested in hearing from all of you in your own words about the successes and challenges, so we can improve academic and community health center partnerships.

***DEFINITIONs***

*Patient-Centered Outcomes Research (PCOR):* PCOR helps people and caregivers communicate and make informed health care decisions, allowing their voices to be heard in assessing the value of health care options and research. Patient-centered care focuses on the relationship between health care providers and patients as equal partners in making decisions.

*Comparative Effectiveness Research (CER)*: CER is about “what works best;” more specifically it identifies what clinical and public health interventions or processes work best for improving health.

***INSTRUCTIONS & GUIDELINES***

We want to remind everyone that there are no right or wrong answers, only different points of view. We are interested in all of your ideas and comments. Please feel free to share your perspective even if it differs from what others have said. You can disagree with one another, and please respect everyone’s opinions. We are interested in knowing what each of you think, so please feel free to be frank and to share your experience, regardless of whether you agree or disagree with what you hear. It is very important that we hear from everyone. Please speak one at a time so that we can listen to your opinions. Keep in mind, we are interested in negative experiences just as we are in positive experiences; and at times the negative experiences are the most helpful.

If it’s OK with you all, we will be audio recording this meeting because we don’t want to miss anything. People often say very important things in these discussions, and we can’t write fast enough to get them all down. The audio recording will be transcribed and destroyed after that. To protect your privacy, we will be on a first name basis. While your name will be said in the recording of this discussion, we will remove it from the transcripts, and it will not be included in any reports that develop from our discussion today. You may be assured of complete confidentiality. We ask that you all respect each other’s confidentiality, and please do not repeat what you hear in this discussion today, even if it seems harmless.

Before we get started, please turn off your cell phones or any electronic device that can make noise. Please feel free to take any needed bio-breaks. If you have questions during the focus group, feel free to let us know at any time. You can also use the chat function in Zoom, if you’d like. What questions do you have at this point? OK, let’s begin.

***QUESTIONS***

1. Firstly, let’s all introduce ourselves (your name and role) and tell us what is bringing you joy in your work?
2. How has COVID-19 had an impact on your work/research and activities?
   1. Impact on health disparities research?
   2. Impact on collaborating with researchers?
   3. What competing priorities have you faced in addressing health equity in COVID-19?
3. What has been successful in any COVID-19 community-research partnerships in the past two year?
   1. What made a difference in your efforts?
4. What were/are some challenges you faced in any COVID-19 community-research partnerships?
   1. Lessons learned that helped or hurt your partnerships or the work?
   2. How did these challenges effect your partnership?
   3. How did you deal with these challenges?
      1. What worked well? What didn’t work?
5. What makes partnerships between academia-researchers and community health center advisors trustworthy?
   1. What can academics/research do to earn the trust of community health center members/advisors?
   2. What do you perceive to be an ideal COVID-19 community-research type of partnership?
6. This next set of question are about your perceptions of research on COVID-19 in your community.
   1. **Patients/community member questions:**
      1. What do you see is a priority for your community regarding health equity in COVID-19?
      2. What kinds of questions do you think are important?
      3. What would motivate you and your community to participate in research?
      4. What types of research partnerships would be valuable?
   2. **Academic researcher questions (& *those who’ve participated in research partnerships*):**
      1. What types of COVID-19 research topics are you most interested in?
      2. What has been your experience with research partnerships with community health centers/clinics?
      3. Who were your partners (academics, community health centers, patients)?
      4. What research did you work on?
   3. **Clinic leadership & staff questions:**
      1. What do you see is a priority for your clinic health equity in COVID-19?
      2. What are the positive things about research partnerships for your clinic(s)?
      3. What are the negative things about research partnerships for your clinic(s)
      4. What questions do you have about involving your advisors/community in research?
      5. How might research partnerships make a difference for your own priorities?
7. What would be helpful for you to do health equity COVID-19 research more efficiently?
   1. What resources or activities do you wish you had (e.g., training)?
   2. What resources or activities do you wish your partners had?
   3. What system/infrastructure/processes changes or support are needed?
8. What assets do you/your organization have to strengthen and support capacity to do COVID-19 health equity research?
   1. Individual, institutional, and community assets
   2. For example, human resources, physical resources, information resources, political resources, and existing intervention resources.

**Supplementary Material 2**

**Representative quotes from study participants informing health equity focused prioritized research topics.**

**Vaccine hesitancy, barriers, misinformation and messaging fatigue** – Participants highlighted issues around vaccine hesitancy and the need to explore what influences patients’ attitudes towards or against vaccination, specifically a need to study groups that refuse to be vaccinated.

“*What are the barriers for people who still are not vaccinated? I think that would be a good thing to figure out and know, because obviously testing is done, we know a lot about COVID, but there is still a subsegment of population who are not vaccinated for various reasons, whether it’s misinformation or their own thought processes. Because the more we can vaccinate, the better it will be fore everybody involved in limiting this disease*.” (CHC leader, interview)

They also acknowledged notable messaging fatigue around COVID in general and booster vaccinations for new variants.

“*Certainly, with the booster, the new bivalent booster shot that we’re administering, I think there are quite a number of people feeling like it’s getting old, [saying] I don’t think I want to get it. I had such a severe side effect. So still a lot of education that’s needed for our community in terms of getting the shots. I think just getting more people vaccinated is our next challenge right now*.” (CHC staff, interview)

A consistent topic for future research and intervention was around misinformation, noting concerns about how to educate and empower the community to recognize misinformation and how to be critical thinkers to decipher or identify misinformation on the internet.

“*We need education, we need workshops, we need culture. And go out and speak and raise our voice that the vaccine, there’s nothing wrong inside of it. To remove all that taboo that the vaccine isn’t sure*.” (Farmworker community member, focus group)

“*Misinformation has become so crucial in COVID-19 and fighting misinformation among minority communities. All minorities are always the last group that gets anything. But what we’ve learned is actually the way misinformation works is they focus on those who aren’t getting a lot of information. So you’re going to have to work there first, not the place where people already get lots of good information. … It’s the constellation of COVID-19, anti-science, anti-women, anti-minority. And I actually feel like working on misinformation in COVID-19 is actually a great way to address all these other issues*.” (Researcher, focus group)

**Mental and behavioral health crisis and impact –** Participants noted COVID-19’s impact on people’s mental health, recognizing many people lost people, which affects individuals and the community at large psychologically and emotionally, and we may not know the extent of impact.

“*One thing we discovered when talking to farmworkers was just a lot of disillusions, a lot of disappointments, a lot of anxiety, depression, lack of work availability, stress being brought on the household. And what we’ve seen is they need an outlet. They needed somebody to talk to. Somebody that wasn’t going to judge them or talk down to them. Mental health is one of those things that impacted our community drastically post-pandemic*.” (Farmworker community leader, focus group)

Additionally, access to behavioral health has been challenging, with a shortage of providers in relation to the need and demand, and require innovative efforts.

“*The county behavioral health, you call them and they say we got nobody right now. And then you call insurance and insurance gives you a list of 5,000 people. None of the have openings and nobody is out there helping families connect with these services. … We have a shortage of behavioral health. So, one of the discussions that I’m having with some colleagues is thinking about how can we develop interventions that don’t necessitate the behavioral health clinician? How do you create community interventions that could be self-sustaining?*” (CHC staff, interview)

Farmworker community leaders also noted barriers to accessing care, including cost (e.g., the need for insurance) and stigma with some communities should be addressed.

“*We need [mental health] programs that will help our community. They don’t help because they would not accept that they need help until we get the stigma out of their minds and tell them you’re not crazy. We all need mental health, and this is how we deal with it. Once we tackle that, I think the rest is easier. … And I always see that need in our community of mental health and teaching that you’re not crazy. There’s ways you deal with these emotions and this is going to help you grow as a person and … in the future your family’s going to be better off*.” (Farmworker community leader, focus group)

**Psychological and educational impact on children and youth** – Participants noted that while children get COVID, the morbidity and mortality has been limited compared to older adults. Nonetheless, it has taken a tremendous toll on their mental health and educational development.

“*We have kids who are anxious, depressed, who have had a hard time readjusting into school life, who are behind academically but the schools are so behind with even doing evaluations and don’t have the staff to support*.” (CHC clinic staff interview)

They also acknowledged the importance of access to behavioral health for children:

“*I think the behavioral health that we have available at our school-based sites is huge. As students are going back to school full time, it’s great for them to understand that behavioral health is available for them to talk to. Because it’s a shock to be off for two years and go back, so just them knowing that through our FQHC we offer those mental health, behavioral health services for them to just drop in, talk to a counselor, talk to a therapist, it’s available for them*.” (CHC leaders focus group)

**Translational research on biological reasons for variation in immune response** – Questions arose around the epidemiology of the virus and variations in immune response, including to vaccines and antiviral therapies for treatment that merit more research.

“…*here we’re handing out these antivirals, and one of the questions is, in this particular slice of the national population, how good are those antivirals working? And then is that preventing hospitalization? Is it preventing long COVID? Is it preventing…rebound? … data that is in the trenches … could be different than the patients that are studied that have primary care at [an academic medical center]. That’s a different patient population*.” (Researcher, interview)

**Challenges within the CHC workforce** – Participants highlighted a need to address the healthcare workforce shortage, and identify real organizational strategies to improve burnout, and planning or establishing infrastructure for sustainable investment.

“… *the healthcare workforce just dwindled. I can tell you on what date our entire staff essentially left. It’s two years later, there’s no relief in sight. It feels like we keep saying we’re going to be done with this and we’re not done, and people just got burnt out and made life decisions about what their priorities were, if they wanted to be closer to family, if they even wanted to be in healthcare. And I would say there was a surprising number of people who left the healthcare field. … especially the nurses, the nurse practitioners and the physician assi*stants.” (CHC leader, interview)

“*Workforce issues remains challenging. That’s across the board due to many things. On the surface everybody knows about burnout and staffing shortages because people are out because of COVID. … I think during COVID there was a lot of shifting in employment, and that was really challenging for the clinics, because they tend to be on the low end of the offering due to the economics of being in the safety net. So we’re still seeing that trickle down. As people shift to these new demands, then that leaves vacancies that are really challenging to fill. … There’s a lot of demand for telecommute now, like remote work, and so that’s challenging on healthcare in general*.” (CHC leader, interview)

**Long COVID and its impact on the medically underserved** – Respondents expressed interest in researching long COVID and the need to explore it as an emerging disparity.

“*Management of unexplained symptoms that may or may not be due to COVID or long COVID. We’ve always had unexplained symptoms in medical care, and now every time someone has an unexplained symptom we’re wondering if it has something to do with COVID. Whether they know they had COVID and they had it or whether maybe even had COVID and didn’t know it and how they have these symptoms, or whether it’s related to the vaccines. There’s tons of questions about how to manage all of the chronic health issues that seem to be emerging after COVID. How does COVID affect things like underlying respiratory conditions or cardiovascular disease*?” (CHC researcher, focus group)

**Optimizing healthcare delivery** – Participants noted the need to optimize healthcare delivery, including telehealth, managing backlogs of preventive care and monitoring chronic conditions. Some specified the importance of exploring the long-term impact of all that was paused. For example, dental care was considered nonessential and noted significant drops in preventative visits that will likely have long term impacts as “*people come back with more severe and serious dental conditions that could have been avoided, if they had kept up with their regular appointments*” (researcher, focus group).

“*I think mammography and PAP for women … because what we might have lost is an opportunity to catch things early. Same thing with colorectal cancer screening. So basically the conditions, both the preventative stuff and the conditions that the bulk of our patients have, diabetes, hypertension, just getting those measures back into our electronic health record and being able to provide more dedicated follow-up on that*.” (CHC staff, interview)

“*I have people I have not been able to get in for care for two years. And people have not gotten follow-up is really scary. Like positive tests that have not been followed up, that in the past would have been because people are terrified to come in. And so I’m really interested in what are we going to do about all the backlog of deferred healthcare? And I hear a lot of patients are doing telehealth, but they won’t come in to get their blood checked. They’re like, I’m not coming into that hospital. It’s full of COVID*.” (researcher, focus group)

Participants also noted a need to explore sustaining the CHC service model and lessons learned to improve response for the next pandemic.

“*I think we should be asking what are the things we should keep, what are the positive things that came out of COVID? I mean, the eternal optimist. Because there are things that we should keep. So even in healthcare, when does the virtual care work well? When should we keep it, when should we not? But remembering not to just focus on the negative piece*.” (Researcher, focus group)

“*How do we have sustainable investment in public health workforce? In building our public health infrastructure to be better responsive, to be more resilient when things like this occur and to be able to like snap into action pretty quickly in order to minimize the harm or damage to patients, to community. And so I’m really thinking about workforce, public health workforce, public health infrastructure and how we can enhance those, prepare the next generation to be equipped to function in a public health setting and how we can build the public health infrastructure to be more response*.” (Researcher, focus group)

**Health outcomes and disparities** – Participants noted the importance to continue examining overall health outcomes and disparities, with a focus on essential workers, diverse populations, social determinants of health, interaction with other chronic conditions, access to vaccines and effectiveness, access to and outcomes of paxlovid/antivirals. Participants expressed concerns regarding equity in COVID testing and vaccination, and differences across various groups – for example rural versus urban and subpopulations like farmworkers or migrant worker populations.

“*I’ve noticed that we need a lot of resources to educate people in the community and also financial support, like support those places like food pantries for them to give more resources to the families who are suffering [with COVID]. They just say hey, you have COVID, go home. They’re losing the day of work, they are losing to be able to pay their bills at home. And that is one of the things that the families suffer. Having the necessary income for rent, food and utilities. It’s something that’s really common and they’re essential*.” (Farmworker community member, focus group)

“*Paxlovid disparities, access to care. It has to be given with three days from the time when you figure out you have something, before you even get diagnosed, right? So there’s a series of access barriers. Recognizing your symptoms, getting a test done, telling your doctor, getting a prescription, going to a pharmacy that actually has the medication. And you could be highly educated and still have trouble getting through that process in three days. And if you’re not highly educated, high resourced, limited English proficient, you can imagine that every one of those things would stretch that out. And I anticipate that we’re going to have huge Paxlovid care disparities*.” (researcher, focus group)

**Trust in healthcare and science** – Trust was a common theme across healthcare and science, particularly around the need to address misinformation. A researcher also noted “COVID did not cause trust issues,” but rather “made it more obvious to the whole community.”

“…*especially around vaccines there was a lot of misinformation. How do we get it out there, how do we talk with them in a way that they will understand? Do they really want to hear from me, an MD, or do they want to hear from a peer, that kind of thing. So there’s no just the collaboration in the healthcare system, but the trust. I think it really amplified the distrust with the medical world in a lot of these communities*.” (CHC staff, interview)

“*No matter how much we try to push information to them, if it’s not, if the information is not elaborated the way that they will digest it, really simple and people that they trust, they will not stop being afraid. Especially with all the misinformation*.” (Farmworker community leader, focus group)

**CHC research participation** – Respondents noted a need for CHCs to participate in more research despite their focus being strictly on clinical care, as research results can help them advance patient care and meet the needs of their patient populations. One participant highlighted the impact of technology and access to smart phones, computers and the internet.

“…*how technology will play into more of this [research] in the future. What we learned phones were so important during COVID and not everyone had a computer but most people had a phone. … I have no idea when it comes to confidentiality with research and how that works*.” (CHC staff, interview)

Participants from farmworker communities acknowledged the importance of participating in research, but noted a great limitation in researchers returning to share results with the community.

“*We would like for you to come back, because for example, when we go to a community and they tell us no, they already came … and then they never came back. So just like that, you leave with everything that we’ve said, but then we don’t know anything from you. So we would like for you to come back, for you to teach us and then we can go out into the streets and do what we’ve always done*.” (Farmworker community leader, focus group)

“*We would like for you to come back and we would like to have answers. Because in all actuality, we need more education, we need more resources. We need more cooperation to be able to be a positive group for the community and for every person who needs us. And for the future pandemics to come*.” (Farmworker community leader, focus group)

Participants also acknowledged when engaging in research to address the workforce shortage and burnout, we must diversify research participation of the workforce.

“*I want this intervention to be one that meaningfully involves a diverse range of healthcare workers. … we know that folks from disadvantaged backgrounds are less likely to be in the physician role, but we have a lot of folks who are like frontline, and they experience higher rates of burnout, so you have to include those types of folks. … For example, parking and transportation workers are having huge turnover. How can we pull in those types of voices that tend to have less power in institutions and tend to be lower wage and just aren’t included in much? So I took this project as an opportunity to say okay, you have a problem with keeping these workers, let’s really focus on that specifically and work to improve equity in your organizations that way, by lifting up voices that perhaps you haven’t listened to in the past*.” (Researcher, focus group)

**Anti-racism** – Participants also brought up anti-Asian hate around COVID that had an impact on how some minority communities were cautious.

“*I think the main issue is probably more on the emotional, psychological impact of COVID, being since the COVID virus originated from China. So, there are still a lot of misunderstanding and hatred, what you call the anti-Asian hate. So, my patients are telling me that at first it’s the virus, now we’re scared to come outside because of the hate crime that’s been going on. So psychologically impact has definitely been [prevalent], especially for the elderly, they see all the videos of people getting beat up or pushed into the train tracks or just killed because they shoved them or kicked them*.” (CHC leader, interview)

**Supplementary Material 3**

**Full list of topics identified during interviews**

**COVID-19 Vaccines**

- How to address vaccine hesitancy and barriers to vaccination in adults.
- How to address vaccine hesitancy and barriers to vaccination in children 0 to 5.
- How to address misinformation about COVID-19 and vaccines.
- What is the relationship between COVID-19 vaccination and infection rates and rates of serious infection, or death.
- How to address misinformation about COVID-19 and vaccines.
- How to address fatigue with COVID-19 messaging (e.g., related to bivalent booster shot).

**Mental and Behavioral Health**

- How to address the mental health crisis caused by COVID-19?
- What are the mental health impacts of COVID-19?

**Translational Research**

- Understanding the biological reasons for different immune responses by different populations to COVID-19 infections.

**Community Health Center Workforce**

- Strategies to address clinician and staff fatigue and burnout.
- How to address workforce shortages?
- How to sustainably invest in a public health workforce?

**Long-COVID**

- Research on Long COVID-19
- impact on health overall
- impact on mental health
- impact on farmworkers
- impact on essential workers
- impact on Latino/a populations

**Outcomes and disparities research**

- How has COVID-19 impacted on the health of essential workers?
- How has COVID-19 impacted on the health of Latino/a populations?
- Explore the relationship between COVID-19 on breathing and allergies in migrant farmworkers.
- How does COVID-19 affect underlying respiratory conditions, cardiovascular disease, and other chronic diseases?
- Relationship between COVID-19 and the social determinants of health.
- Assess the differences in COVID-19 testing resources, vaccination resources, and health outcomes by populations/groups (e.g., urban versus rural, by race/ethnicity, by other social-demographic characteristics, migrant/seasonal workers)
- Research on Paxlovid disparities and access
- Paxlovid and COVID-19 rebounds
- Research on the effectiveness of antivirals on preventing hospitalizations, long-COVID-19

**Children and youth research**

- How do teenagers navigate their post-high school experience following COVID-19?
- Assessing the psychological, behavioral, and emotional health impact of COVID-19 on children and youth.
- How has COVID-19 impacted the educational development/deficit of children and youth?

**Health Care Delivery**

- How to manage all the chronic health issues that seem to be emerging after COVID-19.
- How to optimize telehealth for populations receiving care in community health centers
- How to address the backlog of primary care/preventative health that has been impacted by the pandemic?
- How to integrate COVID-19 information into regular health promotion messages and campaigns?
- How to sustain community health centers so they can continue to provide care to medically underserved populations.
- How to create the public health infrastructure to respond to the next pandemic?
- How to deliver greater access to mental health services?
- How to develop interventions/clinical pathways that are alternatives to behavioral health clinicians? How to create community interventions that could be self-sustaining?
- Developing interventions that support parents
- Strategies to keep everyone safe in clinics due to COVID-19

**Trust in healthcare and science**

- Research to help people recognize and/or identify misinformation on the internet
- How to address the trust deficit between individual patients and communities and healthcare and science

**Research methods**

- How to conduct research in general and related to COVID-19 that includes populations receiving care in community health centers.
- How to improve access to research trials and research results for populations receiving care in community health centers.

**Anti-racism research**

- How to address Anti-Asian hate that has resulted from COVID-19
